# Creutzfeldt–Jakob Disease with Stroke-Like Symptoms: Case report

**DOI:** 10.15388/Amed.2022.29.2.3

**Published:** 2022-06-29

**Authors:** Bünyamin Tosunoğlu, Sıddıka Sena Dilek, Ümmühanı Emektar, Tahir Kurtuluş Yoldaş

**Affiliations:** Ankara Education and Research Hospital, Department of Neurology, Ankara, Turkey; Ankara Education and Research Hospital, Department of Neurology, Ankara, Turkey; Ankara Education and Research Hospital, Department of Neurology, Ankara, Turkey; Ankara Education and Research Hospital, Department of Neurology, Ankara, Turkey

**Keywords:** Creutzfeldt–Jakob disease, stroke, dementia

## Abstract

Creutzfeldt–Jakob disease (CJD) is a rare, progressive, and fatal prion disease. Often the first sign of CJD is rapidly progressive dementia, leading to memory loss, personality changes, and hallucinations. Myoclonus typically occurs in 90% of cases, but often may not be an initial symptom. Other frequently occurring psychiatric symptoms include depression, anxiety, paranoia, obsessive-compulsive symptoms, and psychosis. Speech disorder, loss of balance and coordination may also accompany. We present a case of CJD with sudden onset of right hemiparesis.

## Santrauka.

Creutzfeldto ir Jakobo liga (CJD) yra reta, progresuojanti ir mirtina prioninė liga. Dažnai pirmasis CJD požymis yra greitai progresuojanti demencija, sukelianti atminties praradimą, asmenybės pokyčius ir haliucinacijas. Mioklonusas paprastai būna 90 proc. atvejų, tačiau dažnai tai gali būti ne pirminis simptomas. Kiti dažnai pasitaikantys psichikos simptomai yra depresija, nerimas, paranoja, obsesiniai ir kompulsiniai simptomai bei psichozė. Taip pat gali sutrikti kalba, būti prarasta pusiausvyra ir koordinacija. Pateikiame CJD atvejį, kai buvo staigi dešiniosios hemiparezės pradžia.

## Introduction

Prions are particles that contain only protein, do not have nucleic acid in their structure, can self-replicate, and have a smaller structure than a virus. They cause amyloid deposition in the brain. Prion diseases are called spongioform (spongy) encephalopathies because they cause vacuolization in neurons. Since prions have a structure very similar to normal cell proteins, they do not give antigenic stimulation and do not cause an interferon response. Serological tests are not used for diagnostic purposes [1,2]. Stanley Prusiner in 1982 defined prions as “proteinaceus infectious particles”. Creutzfeldt–Jakob disease (CJD) is a rare, fatal prion disease. The disease is named Creutzfeld–Jakob after the German neurologist Hans Gerhard Creutzfeldt who first described it in 1920, and Alfons Maria Jakob, who described it shortly afterwards [2,3]. 10–15% of CJD cases are familial, 85% are sporadic, and a few are iatrogenic. In the early stage of CJD, personality changes take the form of memory impairment, visual abnormalities, myoclonus [3,4]. In this case study, we present a case of CJD with sudden onset of right hemiparesis.

## Case Report

**Figure 1.**
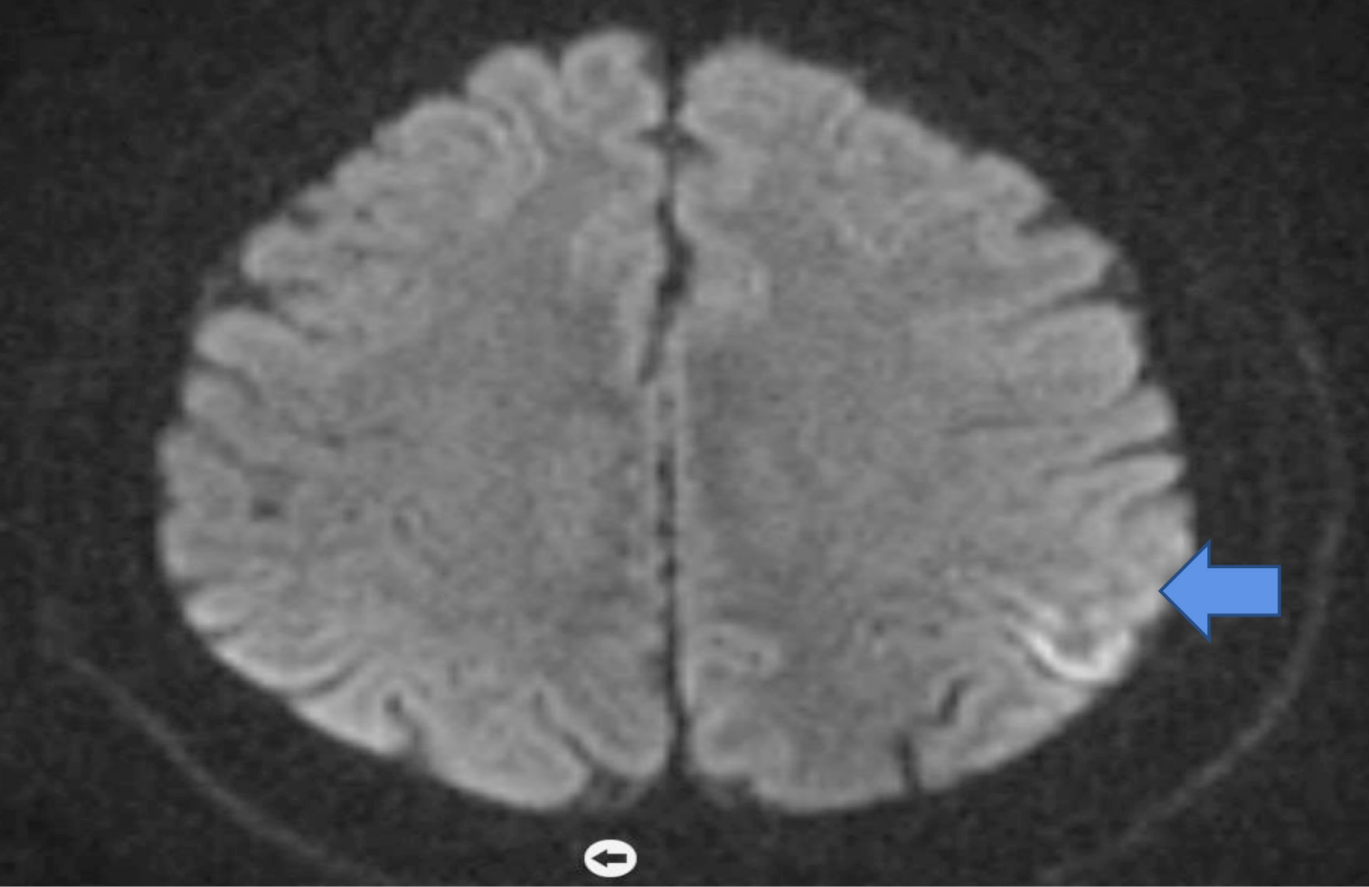
Diffusion-weighted magnetic resonance imaging of the brain

A 64-year-old male patient was brought to our polyclinic by his relatives with the complaints of weakness in the right arm and leg, not being able to recognize his relatives, aggressive behavior, inability to walk, and twitching movements in his hands and arms, which had started a month ago. Then he was hospitalized for a stroke with the complaint of weakness on to his right side, but his relatives stated that the patient did not recognize them, could not walk, exhibited aggressive behavior, and had twitches in his hands and arms for the last month. The first diffusion-weighted brain magnetic resonance (MRI) imaging results were reported as “diffusion limitations suggesting acute-subacute infarction were noted in the cortical structures adjacent to the posterior central sulcus in the left parietal lobe” (Figure 1).

**Figure 2.**
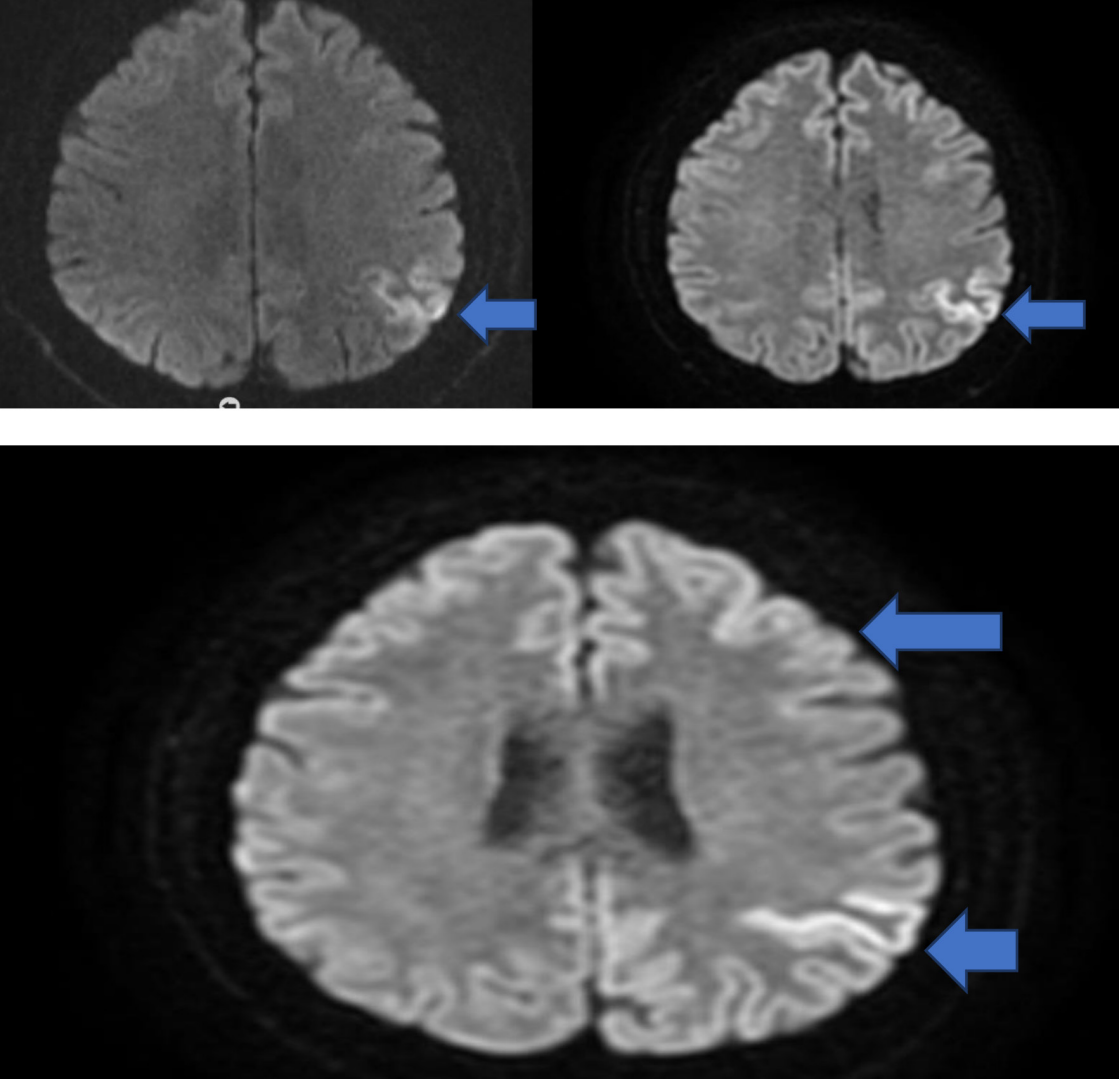
Cortical diffusion restriction in the left parietal region in brain MRI diffusion-weighted imaging.

The patient was using acetylsalicylic acid 300 mg 1x1. He had no other known disease, history of trauma, or substance abuse. He had been a worker at a car plant and retired five years ago. The patient was admitted to the neurology department for further examination, treatment and elucidation of etiology. On physical examination, temperature was 36.6 °C, blood pressure was 130/70 mmHg while lying down, blood pressure was 120/60 mmHg standing up, heart rate was 96/minute, respiration was 18/minute, and oxygen saturation was 95. In his neurological examination, the patient was conscious, but his orientation and cooperation was limited. Pupils were isochoric, light reflex was taken in both eyes and eye movements were free in all directions. The patient had no facial asymmetry and was not communicating. He had 3/5 (Medical Research Council scale) hemiparesis in his right arm and leg on motor examination. He could not cooperate in his sensory and cerebellar examination. The patient was able to stand up with support. Deep tendon reflexes (DTR) were obtained in both upper and lower extremities, plantar response was flexor. There was no sign of meningeal irritation. Although not reliable due to the pateint’s lack of communication and limited orientation and cooperation, his mini mental test score was 16. Routine blood tests, biochemistry, whole blood, vitamin B12 tests, thyroid function tests, copper, ceruloplasmin, HbA1c, erythrocyte sedimentation rate, serum electrophoresis, autoantibody screening (Antinuclear Antibody, anti-SSA, anti-SSB), antithyroid antibodies, syphilis serology (fluorescent treponemal antibody) results were normal. Results of toxicology tests were also within normal limits. Elisa tests (hepatitis A, Hiv, hepatitis B, hepatitis C, rubeola, rubella) were negative. Complete urinalysis results were within normal limits. No agents were found in testing for viral and bacterial meningitis. Results of brucella tests were negative. Serum acetylcholine receptor antibody result was negative. The only abnormalities detected in the lumbar puncture (LP) were cerebrospinal fluid (CSF) protein 1193.4 (normal value 150–450) and Bos immunoglobulin G 140.30 (normal value 0–8.1) values. The oligoclonal bands were negative. CSF sampling for the 14.3.3 protein was positive. No significant pathology was detected in brain computed tomography (CT). In the brain MR diffusion imaging taken after one month, it was reported as “cortical diffusion restriction in the posterior neighborhood of the central sulcus in the left parietal lobe” (Figure 2).

**Figure 3.**
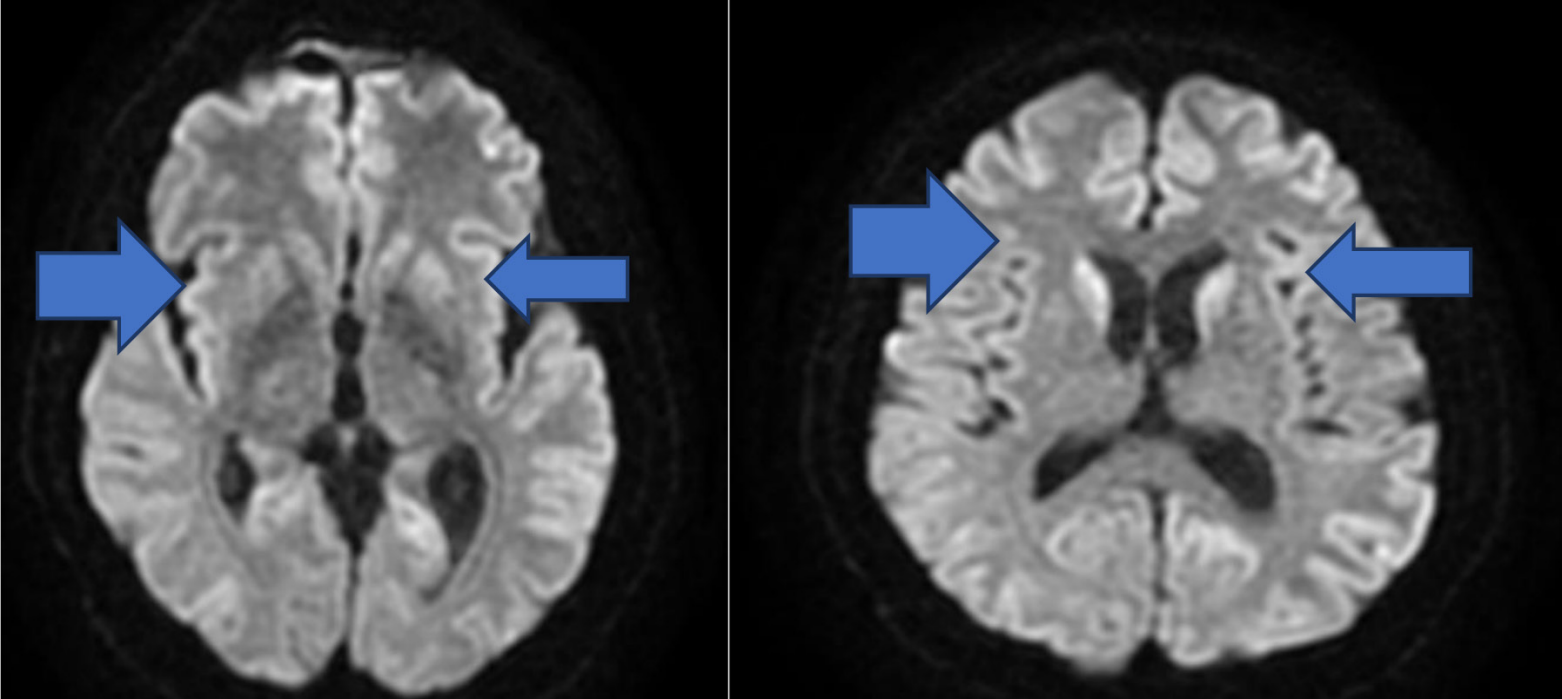
Symmetrical hyperintensity in the anterior putamen and thalamus pulvinar

Signal changes in bilateral putamen and pulvinar on T2-weighted brain MRI suggested CJD (Figure 3).

In the electroencephalography (EEG) examination, it was observed that generalized sharp waves along the trace appeared with occasional periodicity. Conclusions of waking EEG results:

1. Background activity irregularity characterized by moderate frequency slowing.
2. Epileptiform abnormality characterized by generalized sharp waves was observed.

Despite the supportive treatment given the patient’s symptoms progressed rapidly and manifested in akinetic mutism. The patient died on day 21 of hospitalization.

## Discussion

Creutzfeldt-Jakob disease is an almost always a fatal, degenerative disease. Early symptoms include memory problems, personality changes, myoclonus, and vision problems [3]. In our case, the patient initially presented with right hemiparesis and received antiplatelet therapy due to stroke. 70% of patients die within one year of being diagnosed with CJD. Although the sporadic type is the most common variant, it is still very rare. Sporadic cases of CJD occur between the ages of 45 and 75 [4,5]. On average, symptoms appear in age between 60 and 65 years. CJD is a type of contagious spongiform encephalopathy caused by prions. Prions are misfolded proteins that form in central nervous system (CNS) neurons. It is thought to affect signaling processes, damage neurons and cause degeneration in the affected brain resulting in a spongy appearance. The CJD prion is dangerous because it promotes the refolding of the natural prion protein into the diseased state. The number of misfolded protein molecules increases exponentially, leading to large amounts of insoluble protein in the affected cells. These misfolded proteins impair neuronal cell function and cause cell death. Mutations in the prion protein gene occur as a result of misfolding of dominant alpha-helical regions into beta-folded sheets [1]. Transmission has been shown to occur through prion-contaminated human brain products, corneal grafts, dural grafts, or electrodes, implants, and the human growth hormone [4,6]. In differential diagnosis, Alzheimer’s and vascular dementia, Herpes encephalitis, multiple sclerosis, Hashimoto’s encephalitis, Lewy body dementia should be excluded. The diagnosis of CJD has historically been problematic due to the nonspecific nature of early symptoms and the difficulty in safely obtaining brain tissue for confirmation. The diagnosis may be suspected at first in a person with rapidly progressive dementia, especially when they present with characteristic medical signs and symptoms such as myoclonus, difficulties with coordination/balance and walking, and visual disturbances. Further testing results that may support the diagnosis are:

- MR imaging of the brain showing high signal intensity bilaterally, usually in the caudate nucleus and putamen, on T2-weighted images;
- the EEG displaying the characteristic periodic sharp wave pattern;
- 14.3.3 protein positivity in CSF [1,4,5,6].

In our patient, CJD was diagnosed in line with available clinical and imaging studies. Cases of early clinical onset of stroke with subsequent diagnosis of CJD have been reported in the literature. Although there is no specific treatment for CJD, supportive treatment is given.

In summary, our patient presented with right hemiparesis clinic and rapidly progressing dementia. It should not be forgotten that CJD cases can be seen with stroke clinic, as seen in the literature and in our case.
